# Shortages of benzathine penicillin for prevention of mother-to-child transmission of syphilis: An evaluation from multi-country surveys and stakeholder interviews

**DOI:** 10.1371/journal.pmed.1002473

**Published:** 2017-12-27

**Authors:** Stephen Nurse-Findlay, Melanie M. Taylor, Margaret Savage, Maeve B. Mello, Sanni Saliyou, Manuel Lavayen, Frederic Seghers, Michael L. Campbell, Françoise Birgirimana, Leopold Ouedraogo, Morkor Newman Owiredu, Nancy Kidula, Lee Pyne-Mercier

**Affiliations:** 1 Department of Reproductive Health, World Health Organization, Geneva, Switzerland; 2 Division of STD Prevention, Centers for Disease Control and Prevention, Atlanta, Georgia, United States of America; 3 Clinton Health Access Initiative, Boston, Massachusetts, United States of America; 4 Pan American Health Organization, Washington, District of Columbia, United States of America; 5 African Regional Office, World Health Organization, Brazzaville, Congo; 6 Intercountry Support Team for East and Southern Africa, World Health Organization, Harare, Zimbabwe; 7 Bill & Melinda Gates Foundation, Seattle, Washington, United States of America; University of Bern, SWITZERLAND

## Abstract

**Background:**

Benzathine penicillin G (BPG) is the only recommended treatment to prevent mother-to-child transmission of syphilis. Due to recent reports of country-level shortages of BPG, an evaluation was undertaken to quantify countries that have experienced shortages in the past 2 years and to describe factors contributing to these shortages.

**Methods and findings:**

Country-level data about BPG shortages were collected using 3 survey approaches. First, a survey designed by the WHO Department of Reproductive Health and Research was distributed to 41 countries and territories in the Americas and 41 more in Africa. Second, WHO conducted an email survey of 28 US Centers for Disease Control and Prevention country directors. An additional 13 countries were in contact with WHO for related congenital syphilis prevention activities and also reported on BPG shortages. Third, the Clinton Health Access Initiative (CHAI) collected data from 14 countries (where it has active operations) to understand the extent of stock-outs, in-country purchasing, usage behavior, and breadth of available purchasing options to identify stock-outs worldwide. CHAI also conducted in-person interviews in the same 14 countries to understand the extent of stock-outs, in-country purchasing and usage behavior, and available purchasing options. CHAI also completed a desk review of 10 additional high-income countries, which were also included. BPG shortages were attributable to shortfalls in supply, demand, and procurement in the countries assessed. This assessment should not be considered globally representative as countries not surveyed may also have experienced BPG shortages. Country contacts may not have been aware of BPG shortages when surveyed or may have underreported medication substitutions due to desirability bias. Funding for the purchase of BPG by countries was not evaluated. In all, 114 countries and territories were approached to provide information on BPG shortages occurring during 2014–2016. Of unique countries and territories, 95 (83%) responded or had information evaluable from public records. Of these 95 countries and territories, 39 (41%) reported a BPG shortage, and 56 (59%) reported no BPG shortage; 10 (12%) countries with and without BPG shortages reported use of antibiotic alternatives to BPG for treatment of maternal syphilis. Market exits, inflexible production cycles, and minimum order quantities affect BPG supply. On the demand side, inaccurate forecasts and sole sourcing lead to under-procurement. Clinicians may also incorrectly prescribe BPG substitutes due to misperceptions of quality or of the likelihood of adverse outcomes.

**Conclusions:**

Targets for improvement include drug forecasting and procurement, and addressing provider reluctance to use BPG. Opportunities to improve global supply, demand, and use of BPG should be prioritized alongside congenital syphilis elimination efforts.

## Abstract 2

Using country-level surveys and stakeholder interviews, Stephen Nurse-Findlay and colleagues investigate the global frequency of benzathine penicillin shortages and uncover commonly noted causes.

## Author summary

**Why was this study done?:**

- A single dose of low-cost benzathine penicillin G (BPG) ends syphilis infectivity in adults with no documented risk of antibiotic resistance. In spite of this, syphilis continues to infect millions globally.
- Pregnant women with syphilis are particularly vulnerable, as fetal transmission of the infection can cause stillbirth. The only recommended treatment for syphilis in pregnant women is BPG.
- Congenital syphilis remains a significant contributor to early infant mortality, particularly in low- and middle-income countries.
- There are several reasons for this, but one of the most important is a global shortage of BPG.
- In 2015, WHO began to receive anecdotal country reports of BPG stock-outs.
- WHO decided to assess these shortages, describe global supply and demand drivers, and propose viable policy solutions.

**What did the researchers do and find?:**

- The team completed 3 surveys to assess global BPG shortages occurring during 2014–2016.
- The first was distributed to 41 countries and territories in the Americas and 41 African countries.
- The second was emailed to 28 US Centers for Disease Control and Prevention country directors.
- The third was used in in-person interviews in 14 countries by the Clinton Health Access Initiative.
- In all, 95 of 114 unique countries and territories responded to the surveys. Of these, 39 reported a BPG shortage and 56 reported no BPG shortage.
- The team discovered 3 major issues. First, countries often obtain their product from a single wholesaler, which often obtains its products from a single final dose formulator, which often obtains its active ingredient from a single manufacturer. This “sole sourcing,” combined with a highly consolidated market, makes alternative supply difficult if there are production, quality, regulatory, or specification changes within a country’s supply chain.
- Second, as an off-patent medication, BPG commands a market price of pennies per dose. However, as a sterile injectable, it is also expensive to manufacture. These economics have led manufacturers to either abandon BPG production or implement stringent ordering protocols that compromise supply for low- and middle-income countries.
- Third, inaccurate country forecasts, weak procurement systems, and clinical knowledge gaps about syphilis treatment have compromised demand for and procurement of BPG.

**What do these findings mean?:**

- Widespread BPG shortages compromise treatment of adult syphilis, prevention of congenital syphilis, and treatment of other BPG-indicated conditions (including rheumatic heart disease).
- An uninterrupted supply of quality-assured active pharmaceutical ingredient and final formulated product will simplify BPG procurement for high-burden countries.
- Countries must strengthen their supply chain, purchasing, forecasting, and procurement infrastructure to mitigate shortage risk and reduce demand-side stock-outs.
- Countries must also strengthen testing for and treatment of maternal syphilis (and prevention of congenital syphilis) with BPG.

## Introduction

The World Health Organization (WHO) estimates that there are 5.6 million new cases of syphilis annually and 18 million prevalent cases [1]. In 2012, WHO estimated that there were 930,000 pregnant women with syphilis, resulting in 350,000 adverse pregnancy outcomes, with over half of these being stillbirth or neonatal death [2]. Both screening and treatment for syphilis remain suboptimal in low- and middle-income countries (LMICs) [3], despite the diagnosis and prevention of mother-to-child transmission (MTCT) of syphilis being feasible, inexpensive, and cost-effective [4]. Benzathine penicillin G (BPG) is the only recommended treatment for syphilis in pregnant women to prevent MTCT, as other drugs are contraindicated, do not cross the placenta to treat the fetus, or are less effective than BPG [5]. Treatment of syphilis-infected pregnant women with 2.4 million international units (IU) of intramuscular of BPG given at least 28 days prior to delivery can result in an 82% reduced risk of stillbirth and 80% reduction in neonatal mortality [6]. In recognition of this, WHO published *The Global Elimination of Congenital Syphilis*: *Rationale and Strategy for Action* [7] and set country targets for the elimination of MTCT of HIV and syphilis, which will bring BPG shortages into sharp focus.

Syphilis’s causative organism *(Treponema pallidum)* has not developed resistance to first-line treatment with BPG, which is also indicated for secondary prophylaxis of rheumatic heart disease, primary treatment of group A streptococcal pharyngitis, and yaws [5,8,9]. As a result, BPG is considered an essential medicine by WHO [10]; it is typically available as a powder for reconstitution or as a suspension product (marketed in the US, Canada, Australia, New Zealand, and Brazil) [11,12].

As BPG is off patent, it sells for pennies a dose, but as a sterile injectable medication, it is expensive to manufacture. Several active pharmaceutical ingredient (API) manufacturers that make BPG’s active ingredient, and final dose formulators (FDFs) that formulate, package, and label the final product, have stopped producing BPG because of these economics, which has dramatically increased the stock-out risk.

WHO is aware of this vulnerability [13] and advocated a systematic approach to manage these shortages [14]. In May 2016, BPG was recognized by the 69th World Health Assembly as an essential medicine at high risk for stock-out [15]. Despite procurement agency acknowledgment that chronic stock-outs have occurred [14,15], the supply and demand factors that contribute to these shortages have not been previously described.

In 2016, WHO partnered with the Bill & Melinda Gates Foundation and the Clinton Health Access Initiative (CHAI) to (1) identify the extent of BPG stock-outs, (2) determine key contributors to shortages, and (3) propose strategies to mitigate future shortages.

## Methods

### Identifying countries experiencing BPG stock-outs

Data for this analysis were collected from 3 data collection efforts. First, a survey was designed by WHO’s Department of Reproductive Health and Research, adapted and translated from English into Spanish, and distributed in both languages by the Pan American Health Organization (PAHO)/WHO Regional Office for the Americas to the ministries of health of 41 countries and territories (henceforth “countries”) in Latin America and the Caribbean (S1 and S2 Appendices). The same survey was translated into French and used by the WHO Regional Office for Africa (AFRO) in 41 southern, eastern, and western African countries (S3–S5 Appendices). It is important to note that the regional offices were free to adapt the survey based on their needs. AFRO in particular used the survey to also get more information on sexually transmitted infection (STI) management in general, making the answers a bit different from those in the PAHO survey, although the core survey questions were preserved. We also provided the survey electronically, but several respondents to the AFRO survey, and to the US Centers for Disease and Prevention (CDC) survey described below, responded verbally.

Second, WHO conducted an informal email survey of 28 countries (selected because they were PEPFAR [US President’s Emergency Plan for AIDS Relief] countries for which a CDC director was currently assigned) to identify additional countries experiencing BPG shortages or stock-outs (S6 Appendix) (CDC director feedback was normally via email). An additional 13 countries were in contact with WHO for related congenital syphilis prevention activities and also reported on BPG shortages (S7 Appendix).

Third, CHAI developed data collection instruments for in-person interviews in 14 countries (where CHAI has active operations) with BPG distributors, purchasers, ministry of health (MoH) staff, and national leads (S8–S11 Appendices), to understand the extent of stock-outs, in-country purchasing and usage behavior, and available purchasing options.

For countries participating in multiple surveys, a result of BPG stock-out in one survey and no stock-out in another was counted as a stock-out. Countries not involved in these surveys but for whom WHO was in contact regarding issues related to maternal syphilis treatment and congenital syphilis prevention also contributed data on BPG shortages.

In all, 114 countries and territories were approached to provide information on BPG shortages occurring during 2014–2016. Of unique countries and territories, 95 (83%) responded or had information evaluable from public records. A detailed list of sources and indicators used for data collection in these 3 data collection efforts is provided in Table 1.

**Table 1:** Number of countries included in surveys reporting BPG shortage and/or medication substitution for treatment of maternal syphilis.

Source	Number of countries surveyed	Number (%) responded	Number of countries with BPG shortage	Number of countries with medication substitution for BPG	Number of countries with no reported BPG shortage
PAHO survey	41	29 (71%)	5	2	24
AFRO survey	41	35 (85%)	12	1	23
CDC email survey	28	20 (71%)	6	1	14
CHAI survey of LMICs	14	14 (100%)	10	5	4
CHAI desk review of high-income countries	10	10 (100%)	6	NA	4
Additional countries in contact with WHO	13	13 (100%)	4	1	8
Total, with removal of countries included in multiple surveys*	114	95 (83%)	39	10	56

*This represents a summation of the unique countries surveyed.

AFRO, Regional Office for Africa; BPG, benzathine penicillin G; CDC, US Centers for Disease and Prevention; CHAI, Clinton Health Access Initiative; LMICs, low- and middle-income countries; NA, not available; PAHO, Pan American Health Organization; WHO, World Health Organization.

CHAI supplemented the 14 in-depth interviews with a search of grey literature. A systematic review was not completed; however, a snowball technique was employed. CHAI conducted a Google search using a combination of the following terms: “benzathine penicillin” with “brand,” “manufacturer,” or “supplier.” A list of benzathine penicillin names and manufacturers was extracted from the first 5 pages of Google results, which provided a starting list of potential manufacturers and brand names of benzathine penicillin. Duplicates were removed, and a Google search of each brand name with “benzathine penicillin” was conducted.

Relevant information (or lack of a finding) from manufacturer and drug regulatory agency websites was extracted into an Excel database. In addition, specific regulatory databases known to the researchers were searched for “benzathine penicillin.” To validate the hypothesis that stock-outs are a global phenomenon with impact beyond LMICs, CHAI also completed a desk review to scan databases of stock-outs in 10 high-income countries, which were also included in the analysis.

A list of the indicators and sources used in the BPG country surveys is provided in Table 2.

**Table 2:** List of indicators and sources used in BPG country surveys.

Countries	Indicators	Sources
High-income countries (*n* = 10)	• Stock-out reports • Registered product lists	Desk review of National drug regulatory agency databases: • US Federal Drug Administration, European Medicines Agency, Australian Therapeutic Goods Administration, German Federal Institute for Drugs and Medical Devices (BfArM), French National Agency for Medicines and Health Products Safety (ANSM), KNMP Farmanco (Netherlands) Desk review of registered product lists: • API manufacturers: CSPC Pharmaceutical Group (Shijiazhuang, China); Jiangxi Dongfeng Pharmaceutical Company (Leping, China); North China Pharmaceutical Company (Shijiazhuang, China); Sandoz (Kundl, Austria) • FDFs: 12 global FDFs consulted • Wholesalers/distributors: IDA Foundation (Amsterdam, Netherlands); Imres (Lelystad, Netherlands); Missionpharma (Lynge, Denmark); United Nations Children’s Fund
PAHO survey countries (*n* = 41)	• Stock-out reports and reason (all) • Registered product list (Brazil) • Market size (Brazil)	• WHO/PAHO paper survey to MoH on shortages of BPG • Expert interviews with PAHO team • Desk research on Brazil
AFRO survey countries (*n* = 41)	• Stock-out reports	• Paper survey to MoH
CHAI survey countries (*n* = 14)	• Syphilis treatment guidelines • Testing and treatment practices, including barriers and substitutes • Registered product lists • BPG procurement data (volume, trade name, purchaser) • Procurement process • BPG forecast • Stock-out reports and reason	CHAI country teams given standard tool to complete using the following: • Procurement databases • Published guidelines • Interviews with STI program coordinators, clinicians, and MoH or CMS staff with knowledge of registration, procurement, and use of BPG
CDC survey countries (*n* = 28)	• Stock-out reports	• Email survey of CDC country directors

AFRO, Regional Office for Africa; API, active pharmaceutical ingredient; BPG, benzathine penicillin G; CMS, Central Medical Store; CDC, US Centers for Disease and Prevention; CHAI, Clinton Health Access Initiative; FDF, final dose formulator; MoH, ministry of health; PAHO, Pan American Health Organization; STI, sexually transmitted infection; WHO, World Health Organization.

### BPG supply

Supply-side stakeholders were interviewed by CHAI to understand constraints and challenges that may contribute to stock-outs, including capacity, lead times, order patterns, and pricing. CHAI conducted site visits with the 4 API manufacturers, which all operate integrated FDFs currently active in the market.

CHAI requested interviews from 12 FDFs (S12 Appendix) that were purposively selected based on general criteria including available contact information, global reach, and stock-out patterns identified through the survey activities described above. Five FDFs responded to the request for interview, and CHAI was able to conduct semi-structured interviews with all 5. Lastly, CHAI interviewed 4 global wholesalers that sell product to governments.

### BPG demand

The CHAI data collection instrument allowed CHAI to gather country-specific demand-side information on disease prevalence, current clinical practice, product registrations, pricing, historical procurement volumes, and stock-out reports. Interviews were conducted with purchasing agents, national program leads for syphilis, clinicians, and MoH officials, depending on the country. On-the-ground staff in the 14 CHAI countries tailored the data collection methods to the specific country context to collect the requested data. Procurement, pricing, and stock-out data reports were requested and reviewed from the correct procurement or ministry officials. Desk reviews of country-specific treatment guidelines were also conducted.

These inquiries were planned and executed in a stepwise fashion, in the following order: country surveys, supplier interviews, purchasing agent interviews, and clinician and MoH interviews. Each inquiry was informed by results from the previous inquiry. CHAI investigators recorded notes of their discussions by hand.

### Ethical review

The purpose of this assessment was to (1) review BPG availability to prevent and control disease manifestations that represent an immediate risk of untreated or inadequately treated maternal or congenital syphilis, (2) document BPG availability as a public health problem, and (3) improve public health programming. No human research was intended or conducted at the time of collection of these data, and thus this project did not undergo ethical review.

## Results

### Overview of countries experiencing BPG shortages

**Fig 1:**
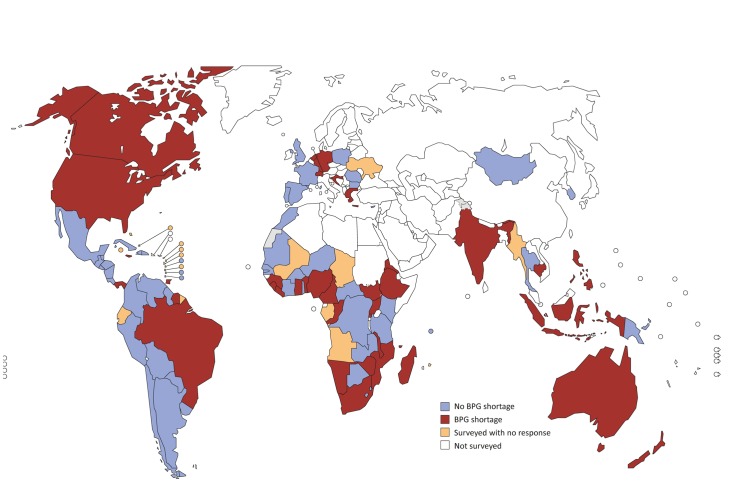
Countries with reported shortages of benzathine penicillin G (BPG) during 2014–2016.

A total of 114 countries and territories were approached to provide information on BPG penicillin shortages or stock-outs occurring during 2014–2016; 24 were part of 2 or 3 of the above-mentioned surveys. After accounting for countries in multiple surveys, 95 (83%) unique countries and territories responded or had evaluable information from public records. Of these 95 countries, 39 (41%) reported a BPG shortage, and 56 (59%) reported no BPG shortage. Ten of these countries reported use of alternative treatments for maternal syphilis including ceftriaxone, amoxicillin, and erythromycin. Three of these countries reported exclusive use of antibiotic alternatives to BPG for treatment of syphilis, and 7 countries reported use of alternatives in addition to reporting BPG shortages (Fig 1; Table 1).

Of the 41 countries and territories in Latin America and the Caribbean surveyed by PAHO, 29 responded (71%). Five reported shortages of BPG (Brazil, Jamaica, Panama, Suriname, and Trinidad and Tobago). Costa Rica reported problems purchasing the 1.2 million UI dose of BPG; Chile reported problems with purchase of the 2.4 million UI dose of BPG. Chile and Nicaragua reported backlogged orders.

Of the 41 countries that received a questionnaire from WHO AFRO to assess availability of BPG, 35 responded (85%), and 12 experienced stock-outs (Benin, Congo, Guinea, Madagascar, Sierra Leone, Eritrea, South Sudan, Comoros, Mozambique, Namibia, Rwanda, and Zimbabwe).

Of the 28 CDC country directors surveyed, 20 (71%) responded, and 6 reported BPG stock-outs (South Africa, Sierra Leone, Namibia, Malawi, Ghana, and Ethiopia).

Of the 14 countries CHAI surveyed to assess availability of BPG, all responded (100%), and 10 reported stock-outs (Cambodia, Cameroon, Ethiopia, Liberia, Malawi, Nigeria, South Africa, Uganda, Zambia, and Zimbabwe).

Thirteen additional countries were in contact, either in person or by phone, with WHO regarding issues related to syphilis. These countries were questioned regarding BPG shortage. Four reported BPG shortage (Philippines, Greece, Croatia, and Switzerland), 1 reported exclusive use of alternative antibiotics for treatment of syphilis (Japan), and 8 reported no shortages.

The study identified a supply shortage of Pfizer’s BPG product in 6 high-income countries (United States, Canada, Australia, New Zealand, Netherlands, and Germany). Large procurement or wholesale agents, such as the United Nations Children’s Fund (UNICEF), were also identified by the CHAI assessment as experiencing challenges sourcing BPG for government and non-profit buyers.

### Supply-side findings

The CHAI assessment demonstrated that, as an older, off-patent formulation, BPG sells at an average of US$0.11 for a 1.2 million IU dose and US$0.20 for a 2.4 million IU dose in LMICs, its largest markets. (This average is based on a CHAI analysis of prices obtained from country-specific procurement data from CHAI BPG country surveys and interviews with distributors.) Additionally, some countries (including India and Brazil) have set a maximum price for BPG, which reduces the commercial attractiveness of this product to API manufacturers.

Significant infrastructure and investment is required to produce a sterile injectable medication like BPG, which limits new market entrants and contributes to supplier exits when production is shifted to less resource-intensive and more commercially attractive products (Box 1). Six API manufacturers and more than 40 FDFs have left the BPG market since the early 2000s.

**Box 1. Country experience with market exits and BPG shortage: Brazil and South Africa**

In 2015, Brazil experienced significant BPG shortages resulting from a combination of market exits and quality disruptions. In 2013, an API manufacturer ended production of pulverized API, 1 of the 2 BPG specifications it produced. FDFs were unable to switch to the API manufacturer’s remaining BPG product, a micronized specification, as it would have required a 2-year production planning cycle, resulted in higher production costs, and not met the need for the specific pulverized product specification.

The leading FDFs in Brazil then required more than a year to find a new API source that matched their required product specifications. They ultimately settled on a Chinese API supplier (previously banned for Good Manufacturing Practice [GMP] issues locally and abroad but already qualified in Brazil for a smaller FDF), which was given a waiver to supply 4 local FDFs with BPG API as a way of stabilizing the country’s supply of the antibiotic. The Brazilian ban due to GMP issues was issued by the Brazilian Health Regulatory Agency (Anvisa) on December 4, 2015, after an inspection, just a month after a noncompliant report from the French National Agency for Medicines and Health Products Safety (ANSM). Seven months later (July 21, 2016), a first waiver was granted, which exempted the API provider from submitting a Drug Master File and allowed it to supply 2 local FDFs with BPG API. A new waiver was granted on November 1, 2016, for the same API provider to supply 2 other FDFs, one of them a local partner of Pfizer [16–18].

However, as both FDFs required time to demonstrate equivalency and receive marketing authorization with the new API supplier, both experienced significant delays to production and market reentry.

The shortage obliged Brazil to issue in 2015 a technical note recommending alternate treatment regimens for non-pregnant women. In its 2016 syphilis epidemiologic bulletin, Brazil reported that the annual rate of congenital syphilis increased from 4.0 cases per 1,000 live births in 2012, to 4.8 in 2013, to 5.4 in 2014, and to 6.5 in 2015. Brazil attributes this increase to several causes, including the BPG stock-outs and misconceptions about BPG adverse outcomes [19].

South Africa exempted the same Chinese API manufacturer from its registry and allowed it to supply over 242,000 vials of unregistered benzathine penicillin to a local manufacturer. The difference in this case is that the Chinese company provided final formulated products, instead of just the API, as was the case in Brazil [20].

Even as higher-margin medications are prioritized in FDF production schedules, 4 of 5 FDFs interviewed noted that the time it takes to switch production lines to manufacture other pharmaceutical products can delay lead times between ordering, production, and delivery for up to 1 year.

**Fig 2:**
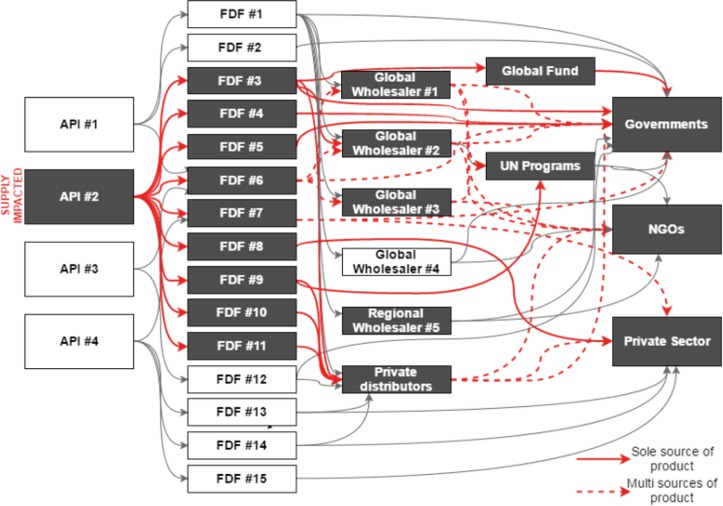
Impact of market shocks on supply in the complex benzathine penicillin market. API, active pharmaceutical ingredient; FDF, final dose formulator; NGO, non-governmental organization.

While API manufacturers have the production capacity to meet global demand, the strategies that they (and FDFs) employ to optimize their commercial position often also constrain supply. For example, minimum purchase order quantities limit the ability of buyers (for example, smaller countries) to make a smaller BPG order. Without pooling of purchase orders across buyers, individual-country demand for BPG is often not enough to meet the minimum purchase order required by API or FDF suppliers. The complexity of the current supply situation and the knock-on effect of changes in any single component of the supply chain are illustrated in Fig 2.

API manufacturer quality is also a concern. None of the 3 API manufacturers currently has market authorization from a stringent regulatory authority for BPG, and 2 of the 3 have experienced GMP quality issues in the past few years [21] (Box 2).

**Box 2. Examples of specific manufacturing disruptions due to quality and regulation issues that have resulted in reduced supply**

In 2014, North China Pharmaceutical Company’s handling of another antibiotic API (procaine penicillin) was considered non-compliant with the European Union (EU) GMP standard. French regulators recommended the withdrawal of the factory’s benzyl penicillin products from the EU market [18]. EU-based FDFs with the North China Pharmaceutical Company API as base (e.g., Panpharma) recalled their products that had been sold to developing countries through distributors, which disrupted supply.

A separate Chinese manufacturer relocated production facilities to a more rural area in order to lower rental costs in 2014. This move required a new GMP certification for API manufacturing, which took a year to obtain. Multiple knock-on effects of this new certification meant that there was a 2-year lag before finished product could be manufactured at their new facilities.

Another issue is that API manufacturers produce APIs with different technical specifications (e.g., particle size and excipient specifications) that adhere to different regulatory constraints. In turn, surveyed FDFs report difficulty in sourcing an alternate “like-for-like” API substitute should their first API manufacturer option go offline for regulatory, quality, or other reasons.

### Demand-side findings

While supply-side delays can significantly impact the availability of BPG, demand-side issues drove stock-outs in 9 of the 10 countries reporting stock-outs surveyed by CHAI; these demand-side issues included (1) poor forecasting, (2) inflexible purchasing cycles, (3) lack of funding, and (4) limited BPG product registrations.

Forecasting inaccuracy resulted in the under-procurement of BPG in 5 of the countries that reported stock-outs to CHAI. Forecasting inaccuracy stems from a lack of product usage data at the facility level. In the absence of facility-level data, countries often rely on historical consumption, purchasing, or prevalence/incidence of BPG-responsive health conditions to estimate how much BPG should be ordered. These data sources may be outdated, be compromised by sub-national differences in incidence or prevalence, be uncorrected for previous stock-outs, be plagued by inaccurate record keeping, or fail to reflect clinician substitution behavior or account for the numerous other bacterial indications for which BPG may be used.

Any combination of these inaccuracies can lead to under-procurement of BPG, as many countries do not have available supplementary budget to purchase buffer stock (a supply of BPG to be held in reserve in case of future supply or demand fluctuations) or additional product. Even when the necessary funds are available, BPG purchasing can be complicated by a procurement system based on annual or 2-year tenders that may also have a minimum purchase order that must be met before fulfillment.

In these cases, even if countries recognize that their original BPG forecast was flawed, these tender processes cannot accommodate orders made mid-cycle, those that request small product volumes, or both. Consequently, countries are often unable to procure product until the next annual tender or an emergency procurement solution is implemented.

The problem is exacerbated when tenders are granted to a single supplier, as the supplier might not have the production flexibility to respond on short notice (Box 3). The study showed that the higher the number of BPG suppliers registered for use in the country, the lower the prospect of a stock-out. Given the lack of a quality-assured BPG product as assessed by a stringent regulatory authority, individual products from individual FDFs must be individually registered for use in each country. If there is a shock to the supplier market, countries without multiple products registered are at higher risk of stock-outs, as they have limited alternatives and must spend considerable time identifying new suppliers and having their products registered for national use.

**Box 3. Country experience with having limited product registration: Cambodia**

Following market exits, Cambodia became reliant on a procurement mechanism that allowed for unregistered, but qualified products to be imported by UNICEF into the country. When Panpharma’s product was recalled, UNICEF was unable to source another qualified brand, and Cambodia’s order went unfulfilled. The country has recently been able to restock its BPG supply.

Having more than 1 registered product can lower the probability of a stock-out. If there is a shock to the supplier market, countries without multiple products registered are at higher risk of stock-outs, as they have limited alternatives and must spend considerable time introducing new suppliers.

Holding buffer stock is one mitigation strategy for supply disruptions. All 6 countries with no buffer stock available or planned had experienced stock-outs, while 3 of the 4 countries that had 3 to 6 months of buffer stock available had not experienced a stock-out in the past 3 years, as this buffer stock allowed them to cover shortages as they arose.

Accordingly, policies to strengthen and improve demand forecasting, in-country purchasing behaviors, and purchasing strategies are required. If these structural issues remain unaddressed and/or unmitigated, demand-side risk of stock-outs may remain even if global supply stabilizes in the next few years.

### Substitutions driving decreased demand

CHAI’s 14-country survey identified decreasing demand for BPG due to the substitution of newer antibiotic classes (cephalosporins, macrolides) in maternal syphilis treatment regimens. Clinically undesirable substitutions are driven by limited BPG availability, intermittent stock-outs, and a lack of BPG knowledge among pharmacists and providers, along with misperceptions of clinical indications, quality, and the possibility of adverse outcomes.

**Fig 3:**
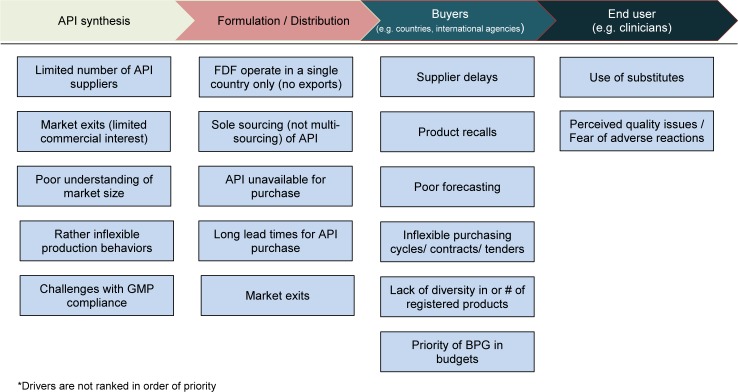
Identified drivers of BPG stock-outs across the value chain. *API, active pharmaceutical ingredient; BPG, benzathine penicillin G; FDF, final dose formulator; GMP, Good Manufacturing Practice.

A summary of all the supply and demand elements of this global shortage is shown in Fig 3, and select responses from providers describing barriers to administration of BPG are provided in Table 3.

**Table 3:** Select responses from providers describing barriers to administration of BPG.

Barrier	Select responses
Pain	“The pain experienced by patient can prevent the use of the drug.”—ZAMBIA “Resistance by injection room health workers to administer BPG as afraid of problem during administration like blocking of needles and not dispersing during mixing with water.”—ETHIOPIA “[Barriers are] pain and that quality of medicine is bad”—UGANDA *(“Pain” also cited as a barrier by Cameroon*, *Malawi*, *and Zimbabwe)*
Anaphylactic shock	“A belief that it kills patients when using it. Some think it might be from using poor quality BPG products.”—ETHIOPIA “In southern state of Tamil Nadu and Kerala, BPG was banned [after adverse event] in terms that it was supposed to be only administered in hospitals but the word that spread was that government had [completely] banned BPG.”—INDIA *(“Risk of anaphalactic shock” also cited as a barrier by Cambodia*, *Cameroon*, *Indonesia*, *Malawi*, *Nigeria*, *Uganda*, *and Zimbabwe)*
Availability of substitutes	“3rd generation antibiotics are more profitable and available; we are moving from injections to orals”—NIGERIA “Would rather use more available cephalosporins, which work against multiple infections”—TANZANIA “[Providers] like ability to use broad based antibiotic”—MALAWI *(“Availability of substitutes” also cited as a barrier by Ethiopia*, *Indonesia*, *and Nigeria)*
Lack of awareness of BPG use for maternal syphilis treatment	**“**Significant number of health workers belief BPG is outdated medicine”—ETHIOPIA **“**There is a training gap of health workers on diagnosis, treatment & management”—LIBERIA **“**Top pharmacist was surprised we asked about BPG. He thought it was an obsolete drug”—NIGERIA *(“Lack of awareness of product indications” also cited as a barrier by India)*
Lack of available BPG	“Artificial stock-outs at facilities exist even when CMS [Central Medical Store] has product”—ZAMBIA *(“Lack of available BPG at facility level” also cited as a barrier by Cambodia*, *Indonesia*, *Nigeria*, *South Africa*, *Uganda*, *and Zimbabwe)*

BPG, benzathine penicillin G.

CHAI’s survey identified 3 countries where BPG was not the recommended first-line treatment for adult syphilis in clinical guidelines and 2 additional countries where BPG was not the most widely prescribed treatment for maternal syphilis despite clear guideline recommendations. Substitutions for adult, and specifically maternal, syphilis treatment were highlighted during interviews in 4 additional countries.

Although not specifically asked in the questionnaire, 3 countries in CHAI’s survey voluntarily reported that BPG demand was limited because many pregnant women were not being tested for syphilis during antenatal care visits. This was related to the limited availability of diagnostics, particularly low-cost rapid diagnostic tests, as well as a continued reliance on lab-based diagnostics, which often do not return results in a timely fashion.

## Discussion

In this evaluation, 39 countries of 95 (41%) responding to surveys reported shortages or stock-outs of BPG occurring during 2014–2016 (114 countries/territories surveyed). Both high-income countries and LMICs were affected.

Overall, the BPG market suffers from limited transparency across a fragmented landscape of API manufacturers, FDF suppliers, procurement agents, and local buyers. On the supply side, BPG presents an unattractive business case as it is an older, off-patent, sterile injectable drug that is expensive to make but commands a market price of pennies per dose. This has led to numerous market exits and the adoption of stringent margin optimization strategies by suppliers, such as inflexible production cycles and minimum order quantities. On the demand side, inaccurate forecasts and suboptimal purchasing strategies, such as inflexible purchasing cycles and sole sourcing of supply, lead to under-procurement of BPG.

This is the first comprehensive assessment to our knowledge to describe (1) the scope of BPG shortages at the national and procurement level, (2) the supply and demand drivers for this essential antibiotic, (3) relevant systemic factors leading to shortages, and (4) viable policy solutions to address the issue. Building on relevant reports such as the *Global Status of BPG Report* [22], this analysis clarifies how shortages of this drug equally affect developing and developed countries, and its detailed insights into suboptimal forecasting and the clinical use of BPG are also valuable for understanding how reduced demand impacts the global supply of this drug.

This study has limitations. The assessment was based on a convenience sample of countries, and of sources from participating countries, and should not be considered globally representative. Other countries may be experiencing BPG shortages that were not included in our study. Persons contacted during the assessment may not have been aware of BPG shortages at the time of the survey. Underreporting of substitution behaviors may have occurred due to desirability bias. Funding for the purchase of BPG by countries was not evaluated.

On the supply side, increasing transparency between the market players, and harmonizing product quality and specification standards across major buyers, is needed. A common product specification accepted by all major stakeholders would lower costs and ease administration for suppliers; it would simplify product substitution of registered and approved API manufacturers and FDFs should any single supplier discontinue operations for any reason, while decreasing supply uncertainty and product variability for buyers throughout the supply chain.

Accordingly, in December 2016, WHO applied for BPG to be listed as a “prequalified” medication [22] and invited API manufacturers and FDFs to apply for WHO prequalification. The WHO Prequalification of Medicines Programme helps ensure that medicines supplied by international procurement agencies such as UNICEF, the United National Population Fund, the Global Fund to Fight AIDS, Tuberculosis and Malaria, and Unitaid meet acceptable standards of quality, safety, and efficacy [23]. This designation can also establish manufacturing standards on particle size and dissolvability that could align the major producers of APIs and FDFs.

From a demand perspective, there must be twin policy emphases. The first is to strengthen the infrastructure that supports national-level BPG forecasting and procurement as a health system priority. When faced with a stock-out, many countries do not have available funds to purchase buffer stock or additional product. Even when necessary funds are available, many countries are constrained by inflexible annual tender processes that cannot accommodate small mid-cycle purchases. There is a need to generate awareness among country procurement agencies and provide technical assistance to help countries assess their need for BPG across the complete spectrum of BPG-treatable pathologies [22] (including primary prevention of rheumatic fever; treatment of pyoderma, yaws, bejel, and pinta; prophylaxis in sickle cell patients following splenectomy; and prophylaxis of recurrent cellulitis) to avoid stock-outs due to forecasts based on syphilis prevention alone. There is a need to generate awareness among country procurement agencies to preemptively mitigate stock-outs via increased product registration, buffer stock management, and rationing procedures to protect against supply disruptions and cover shortages as they arise.

The second policy emphasis should focus on supporting clinical testing and appropriate treatment of syphilis as a public health priority. Clinicians may incorrectly select broader spectrum antibiotics that can treat multiple infections but are less effective against syphilis. This is particularly important in pregnant women, where only BPG is known to cross the placenta barrier and prevent congenital syphilis [5,24,25].

Clinicians may make these incorrect treatment choices due to misperceptions of BPG’s clinical indications, quality, or likelihood of adverse outcomes. Penicillin-related anaphylaxis only occurs in approximately 1 patient per 100,000 administrations (range 0 to 3) [26]. Clinicians may equate even anecdotal reports of adverse events with a perception of a poor-quality drug product, which limits uptake of BPG, further decreases demand, and compromises procurement efforts.

If these structural issues remain unaddressed and/or unmitigated, demand-side risk of stock-outs may remain even if global supply stabilizes in the next few years.

Misperceptions of healthcare providers regarding the clinical indications for use of BPG were addressed by Argentina and Brazil in relation to these shortages affecting congenital syphilis prevention [27,28]. Retraining of healthcare workers on the WHO-recommended treatment of syphilis with BPG [5] and the technique for administration of BPG may be necessary to increase use. Reintroduction of BPG as a component of syphilis testing and treatment into national strategies and guidelines, particularly for maternal and neonatal health, among other health conditions for which WHO recommends the use of BPG, may also increase provider use and demand.

In 2014, WHO published the *Global Guidance on Criteria and Processes for Validation*: *Elimination of Mother-to-Child Transmission of HIV and Syphilis* [29]. Country progress towards achievement of validation indicators has led to increased demand for syphilis treatment and has uncovered several BPG supply chain gaps leading to medication shortages in low-, middle-, and high-income countries, many with a high burden of syphilis. Opportunities to improve global supply, demand, and use of BPG should be prioritized alongside congenital syphilis elimination efforts.

In summary, this assessment represents the first comprehensive analysis, to our knowledge, of the supply and demand drivers for the global shortage of BPG. The global targets for congenital syphilis elimination will not be met until this global BPG shortage is addressed both from a supply and demand perspective. Viable policy approaches to both strengthen procurement infrastructure and support the appropriate treatment of syphilis at the national level are needed.

## Supporting information

S1 Appendix WHO PAHO BPG country survey (English). (DOCX)

S2 Appendix WHO PAHO BPG country survey (Spanish). (DOCX)

S3 Appendix WHO AFRO STI country survey (English). (DOCX)

S4 Appendix WHO AFRO STI east and southern country survey (English). (DOCX)

S5 Appendix WHO AFRO STI country survey (French). (DOCX)

S6 Appendix WHO email survey used for CDC country directors. (DOCX)

S7 Appendix List of countries surveyed by WHO region. (DOCX)

S8 Appendix CHAI’s interview guides: Distributors. (DOCX)

S9 Appendix CHAI’s interview guides: Purchasers, MoH staff, national leads. (DOCX)

S10 Appendix CHAI’s interview guides: Manufacturers. (DOCX)

S11 Appendix CHAI BPG root cause analysis data collection tool. (XLSX)

S12 Appendix List of FDFs contacted by CHAI. (DOCX)

## Abbreviations

AFRO: Regional Office for Africa
API: active pharmaceutical ingredient
BPG: benzathine penicillin G
CDC: US Centers for Disease and Prevention
CHAI: Clinton Health Access Initiative
FDF: final dose formulator
GMP: Good Manufacturing Practice
IU: international units
LMICs: low- and middle-income countries
MoH: ministry of health
MTCT: mother-to-child transmission
PAHO: Pan American Health Organization
STI: sexually transmitted infection
UNICEF: United Nations Children’s Fund
WHO: World Health Organization
