# Supplementary material for: Shortages of benzathine penicillin for prevention of mother-to-child transmission of syphilis: An evaluation from multi-country surveys and stakeholder interviews
Source: PLoS Med. 2017 Dec 27;14(12):e1002473. doi: 10.1371/journal.pmed.1002473 (PMC5744908; doi:10.1371/journal.pmed.1002473)
Supplement: S1 Appendix — (DOCX) [file pmed.1002473.s001.docx]

| **Status of Benzathine Penicillin supply in countries** |
| --- |

**Projected country need for Benzathine Penicillin (BenPCN) to for treatment of maternal syphilis**

These questions are designed to evaluate shortages of benzathine penicillin. This formulation of penicillin is used for the treatment of pregnant women with syphilis as it is the only known effective treatment to prevent congenital syphilis. Responses from this survey will be used to advocate for improvements in benzathine penicillin supply.

Please complete this survey and submit your findings no later than 18 January 2016.

**NEXT**

**Benzathine Penicillin stock out status**

1. Is there a BenPCN stock out at the moment in your country (at the central level)?

( ) NO

( ) YES

If NO, what are the total usable number of doses currently in storage in country (1 dose = 2.4 million IU?

If YES, since when?

**PREVIOUS NEXT**

**Status of Benzathine Penicillin backlogs**

2. Does your country have any backlogged orders?

( ) NO

( ) YES

If yes, what are the numbers of doses for all backlogged orders?

If yes, what are the expected delivery dates?

**PREVIOUS NEXT**

**Procurement mechanisms for Benzathine Penicillin**

3. What mechanisms does your country regularly use to acquire BenPCN? [Mark all that apply, and use the box below for more detailed answers]

( ) There is no centralized acquisition of BenPCN in my country

( ) National bid

( ) International bid

( ) Procurement agreements with a UN Agency

Please specify which UN agency is used for procurement, or share any other relevant comments on this issue in the space below:

**PREVIOUS NEXT**

**Alternative procurement mechanisms used to manage shortage**

4. If there is (or was) a shortage in your country, has your country used alternative mechanisms to acquire BenPCN? [Mark all that apply, and use the box below for more detailed answers]

( ) There is no shortage of BenPCN in country

( ) NO, my country has NOT used alternative mechanisms to acquire BenPCN

( ) YES, my country HAS used alternative mechanisms to acquire BenPCN

If YES, which alternative mechanisms to acquire BenPCN have been used in your country?

**PREVIOUS NEXT**

**Average monthly consumption**

5. What has been the average monthly consumption of BenPCN doses over the past year in your country?

6. Based on your country's projected need for BenPCN and current supply, what is the anticipated BenPCN shortfall in total number of doses for 2016?

**PREVIOUS NEXT**

**Estimated need to treat pregnant women**

7. If available, what is the estimated number of doses needed to treat pregnant women with syphilis in 2016?

**PREVIOUS NEXT**

**Insight for the shortage of BenPCN**

8. Please provide insight as to whether any of the following reasons for a shortage of BenPCN applies in your country. [Mark all that apply, and use the box below for more detailed answers]:

( ) There is no shortage of BenPCN in country

( ) There is a backlog of orders (high demand)

( ) Increased demand for BenPCN beyond current ordering history or allowance

( ) Funding not available in country for purchase of BenPCN

( ) No manufacturer available for purchase

( ) No distributers available for purchase

Are there any reasons not mentioned above for the BenPCN shortage in your country? (Please specify):

**PREVIOUS NEXT**

**Other relevant information on BenPCN shortages**

9. If your country is experiencing BenPCN shortage, please provide insights to the following questions in the box below to help WHO/PAHO better understand the problem:

9a. Please list the issues your country is facing due to the shortage (i.e. purchased BenPCN at higher cost; in different presentation; others):

9b. Please list the perceived causes for BenPCN shortage:

9c. Please list actions taken thus far by the country to minimize problems due to the BenPCN shortage:

9d. Please list desired solutions for BenPCN shortage:

**PREVIOUS NEXT**

**Respondent contact details**

10. Please provide contact details of the person responsible for completing the survey:

Name:

Position:

Institution:

Country :

e-mail:

phone:

**PREVIOUS DONE**
