# Supplementary material for: Shortages of benzathine penicillin for prevention of mother-to-child transmission of syphilis: An evaluation from multi-country surveys and stakeholder interviews
Source: PLoS Med. 2017 Dec 27;14(12):e1002473. doi: 10.1371/journal.pmed.1002473 (PMC5744908; doi:10.1371/journal.pmed.1002473)
Supplement: S2 Appendix — (DOCX) [file pmed.1002473.s002.docx]

**Evaluación de la disponibilidad de Penicilina benzatinica (BenPCN) en los países**

**Necesidad del país para Penicilina benzatinica para el tratamiento de sífilis en mujeres embarazadas**

Estas preguntas están diseñadas para evaluar la disponibilidad de Penicilina benzatinica. La formulación de Penicilina benzatinica se utiliza para el tratamiento de sífilis en mujeres embarazadas, ya que es el único tratamiento, del que se conoce, eficaz para la prevención de la sífilis congénita. Las respuestas de esta encuesta serán utilizadas para abogar en la mejora del abastecimiento de penicilina benzatinica.

Por favor envíe sus respuestas, a más tardar, el día 18 de enero de 2016.

**PRÓXIMO**

**Desabastecimiento de Penicilina benzatinica**

1. ¿Existe actualmente desabastecimiento de BenPCN en su país (a nivel central)?

( ) NO

( ) SÍ

Si no, ¿cuántas dosis utilizables de BenPCN están almacenadas actualmente en el país (1 dosis= 2,4millones de UI)?

Si sí, ¿desde cuándo?

**ANTERIOR PRÓXIMO**

**Compras atrasadas de Penicilina benzatinica**

2. ¿Tiene el país alguna solicitud de compra de BenPCN atrasada? (Por favor, utilice el cuadro de abajo por su respuesta)

( ) NO

( ) SÍ

Si sí, ¿cuál es número total de dosis de BenPCN atrasadas?

Si sí, ¿cuáles son los plazos de entrega previstos para estas solicitudes?

**ANTERIOR PRÓXIMO**

**Mecanismos de adquisición de BenPCN**

3. Que mecanismos de adquisición utiliza regularmente su país para la compra de BenPCN [Marque todo lo que corresponda, y utilice el cuadro de abajo para dar más detalles]:

( ) El país no utiliza adquisición centralizada de BenPCN

( ) Licitación nacional

( ) Licitación internacional

( ) Acuerdos de contratación con una agencia de las Naciones Unidas

Si hay acuerdos de contratación con una agencia de las Naciones Unidas, por favor describir cual agencia y otros mecanismos no mencionados anteriormente en el cuadro de abajo:

**ANTERIOR PRÓXIMO**

**Mecanismos alternativos de adquisición de BenPCN**

4. Como consecuencia de la falta de disponibilidad de BenPCN, ha utilizado el país otros mecanismos alternativos para adquirir BenPCN? [Marque las opciones que apliquen, y utilice el cuadro de abajo para dar más detalles]

( ) No existe escasez de BenPCN en el país

( ) NO, el país NO ha utilizado otros mecanismos alternativos para adquirir BenPCN

( ) SÍ, el país HA utilizado

Si sí, ¿Cuál(es) mecanismos alternativos el país ha utilizado para adquirir BenPCN?

**ANTERIOR PRÓXIMO**

**Consumo promedio mensual**

5. ¿, Cuál ha sido consumo promedio mensual a nivel nacional de BenPCN durante el último año en su país?

6. ¿Con base en las necesidades proyectadas y el abastecimiento actual de BenPCN en el país, cuál es la demanda prevista para el 2016, de BenPCN, expresada en número total de dosis?

**ANTERIOR PRÓXIMO**

**Estimación para tratar a mujeres embarazadas**

7. Si está disponible, cuantas dosis estima serán necesarias para tratar a mujeres embarazadas con sífilis en el 2016?

**ANTERIOR PRÓXIMO**

**Posibles razones del desabastecimiento de Penicilina benzatinica**

3. Favor indicar cuales son las posibles razones del desabastecimiento aplican en su país [marque las opciones que apliquen, y utilice el cuadro de abajo para dar más detalles]:

( ) No hay desabastecimiento de BenPCN en el país

( ) Existe atraso de solicitudes de compra (alta demanda)

( ) Aumento en la demanda de BenPCN en el país superior a lo solicitado o autorizado históricamente

( ) El país no dispone de financiamiento para la compra de BenPCN

( ) No hay disponibilidad de fabricantes para comprar BenPCN

( ) No hay disponibilidad de distribuidores para la compra de BenPCN

¿Hay otras razones no mencionadas anteriormente para el desabastecimiento de Penicilina benzatinica en el país? (Por favor describir)

**ANTERIOR PRÓXIMO**

**Otras informaciones sobre la problemática en el país**

8. Si existe (o existió) desabastecimiento de BenPCN en el país, por favor responda a las siguientes preguntas en el cuadro de abajo, las cuales orientaran a la OPS/OMS para entender mejor la problemática:

( ) ¿Qué dificultades enfrenta el país debido al desabastecimiento de BenPCN (por ejemplo, la adquisición de BenPCN a un precio mayor; en diferentes presentaciones, otras)?

( ) Indique en su opinión cuales son las razones del desabastecimiento de BenPCN en el país:

( ) Por favor indique las medidas tomadas hasta ahora por el país para minimizar los problemas debido al desabastecimiento de BenPCN:

( ) Por favor describa que soluciones serían deseables para el país a fin de afrontar el desabastecimiento de BenPCN:

**ANTERIOR PRÓXIMO**

**Persona responsable que completó la encuesta**

Le solicitamos informar los datos de la persona responsable que completó la encuesta:

Nombre:

Cargo:

Institución:

País:

Correo electrónico:

Teléfono:

**ANTERIOR TERMINADO**
