# Supplementary material for: Shortages of benzathine penicillin for prevention of mother-to-child transmission of syphilis: An evaluation from multi-country surveys and stakeholder interviews
Source: PLoS Med. 2017 Dec 27;14(12):e1002473. doi: 10.1371/journal.pmed.1002473 (PMC5744908; doi:10.1371/journal.pmed.1002473)
Supplement: S3 Appendix — (DOCX) [file pmed.1002473.s003.docx]

**Survey on the status of management of Sexually Transmitted Infections (STIs), including congenital syphilis and availability of Benzathine Penicillin in African countries**

**Background:**

Focal persons from your country in charge of Maternal Health and management of STIs in the Ministry of Health and in WHO offices are invited to participate in a regional workshop for dissemination of WHO newly released (2016) guidelines for management of sexually transmitted infections and antenatal care, which will take place in Ouagadougou, Burkina Faso, 15-17 November, 2016. In order to support the implementation of these guidelines, we would gratefully appreciate the participation of your country in this pre-workshop survey, by answering the following questions. For each question, please feel free to add any comments if necessary.

Grateful if you could complete this survey and submit your responses as soon as possible, but no later than 11 November 2016 to the following persons:

Dr Ouedraogo Leopold: [ouedraogol@who.int](mailto:ouedraogol@who.int); Dr Bigirimana Françoise: [bigirimanaf@who.int](mailto:bigirimanaf@who.int); and Dr Sanni Saliyou: [sannis@who.int](mailto:sannis@who.int)

**Part A: Management of STIs**

This part is designed to assess the status of the planning, implementation, and monitoring of STI programmes in general.

**Burden of ISTs:**

- Total cases in 2015 (all types of STIs)
- Total cases by type of STIs in 2015
- Number of pregnant women tested positive for Syphilis during ANC in 2015

**Policy:**

1. Existence of national strategy for prevention and control of STIs, possibly included in the Reproductive Health (RH) or HIV strategy

( ) NO

( ) YES

1. Existence of national guidelines for management of STIs?

( ) NO

( ) YES

If yes, indicate the year of the last update

1. Existence of national policy documents/ guidelines on prevention and management of congenital syphilis

( ) NO

( ) YES

If yes, indicate the document and the year of the last update

**Implementation strategy:**

1. Is there a functional monitoring and evaluation system in place for STIs?

( ) NO

( ) YES

1. Are monitoring data integrated in the HMIS?

( ) NO

( ) YES

1. Is Syphilis considered in the focused antenatal care package?

( ) NO

( ) YES

**Health workers capacity**

1. Are pre-service and in-service training on-going for prevention and control of STIs? (please precise)

( ) NO

( ) YES

1. Existence of training materials for prevention and control of STIs?

( ) NO

( ) YES

If yes, indicate available training materials, the dates of trainings and number of health workers trained in 2015-2016

1. Is a supervision system in place for prevention and control of STIs?

( ) NO

( ) YES

**Procurement and supply chain:**

1. Availability of essential drugs for management of STIs?

( ) NO

( ) YES

1. Availability of syphilis test kits?

( ) NO

( ) YES

1. Is the country using the dual test for HIV and syphilis for pregnant women?

( ) NO

( ) YES

**Part B: Management of congenital syphilis and availability of Benzathine Penicillin**

This part is designed to evaluate shortages of syphilis test kits and benzathine penicillin (BenPCN). This formulation of penicillin is used for the treatment of pregnant women with syphilis as it is the only known effective treatment to prevent congenital syphilis. Responses from this survey will be used to advocate for improvements in dual test kit and benzathine penicillin supply.

**Benzathine Penicillin stock out**

1. Does BenPCN available in the country?

( ) NO

( ) YES

If yes, who is providing resources for supply of BenPCN?

( ) Government

( ) Partners (please indicate which partners)

2. Is there a BenPCN stock out at the moment in your country (at the central level)?

( ) NO

( ) YES

If YES, since when?

If NO, what are the total usable number of doses currently in storage at the central level (1 dose = 2.4 million IU?)

**Status of Benzathine Penicillin backlogs**

3. Does your country have any backlogged orders?

( ) NO

( ) YES

If yes, what are the numbers of doses for all backlogged orders?

If yes, what are the expected delivery dates?

**Procurement mechanisms for Benzathine Penicillin**

4. What mechanisms does your country regularly use to acquire BenPCN? [Mark all that apply, and use the box below for more detailed answers]

( ) There is no centralized acquisition of BenPCN in my country

( ) National bid

( ) International bid

( ) Procurement agreements with a UN Agency

Please specify which UN agency is used for procurement, or share any other relevant comments on this issue in the space below:

**Alternative procurement mechanisms used to manage shortage**

5. If there is (or was) a shortage in your country, has your country used alternative mechanisms to acquire BenPCN? [Mark all that apply, and use the box below for more detailed answers]

( ) There is no shortage of BenPCN in country

( ) NO, my country has NOT used alternative mechanisms to acquire BenPCN

( ) YES, my country HAS used alternative mechanisms to acquire BenPCN

If YES, please indicate which mechanisms

**Average monthly consumption**

6. What has been the average monthly consumption of BenPCN doses in 2015 in your country?

7. Based on your country's projected need for BenPCN and current supply, what was the estimated BenPCN shortfall in total number of doses for 2016?

**Estimated need to treat pregnant women**

8. If available, what was the estimated number of doses needed to treat pregnant women with syphilis in 2016?

**Insight for the shortage of BenPCN**

9. Please provide insight as to whether any of the following reasons for a shortage of BenPCN applies in your country. [Mark all that apply, and use the box below for more detailed answers]:

( ) There is no shortage of BenPCN in country

( ) There is a backlog of orders

( ) Exceptional increased demand for BenPCN

( ) Funding not available in country for purchase of BenPCN

( ) No manufacturer available for purchase

( ) No distributers available for purchase

Are there any reasons not mentioned above for the BenPCN shortage in your country? (Please specify):

**Other relevant information on BenPCN shortages**

10. If your country is experiencing BenPCN shortage, please provide insights to the following questions in the box below to help WHO/AFRO better understand the problem:

10a. Please list the issues your country is facing due to the shortage (i.e. purchased BenPCN at higher cost; in different presentation; others):

10b. Please list the perceived causes for BenPCN shortage:

10c. Please list actions taken by the country to minimize problems due to the BenPCN shortage:

10d. Please list desired solutions for BenPCN shortage:

**Any other comments or suggestions**

**11. Please provide any additional comments to improve the STIs management.**

For example, we will be interested to hear about the acceptability and utilization of BenPCN.

**Respondent contact details**

12. Please provide contact details of the person responsible for completing the survey:

Name:

Position:

Institution:

Country:

e-mail:

Phone:
