# Supplementary material for: Shortages of benzathine penicillin for prevention of mother-to-child transmission of syphilis: An evaluation from multi-country surveys and stakeholder interviews
Source: PLoS Med. 2017 Dec 27;14(12):e1002473. doi: 10.1371/journal.pmed.1002473 (PMC5744908; doi:10.1371/journal.pmed.1002473)
Supplement: S4 Appendix — (DOCX) [file pmed.1002473.s004.docx]

**Survey on the status of management of Sexually Transmitted Infections (STIs), including for prevention of congenital syphilis and availability of Benzathine Penicillin in East and Southern African countries**

**Background:**

The WHO Africa Regional Office in collaboration with WHO HQ is planning an inter-country workshop for orientation and dissemination of newly released WHO GUIDELINES FOR MANAGEMENT OF SEXUALLY TRANSMITTED INFECTIONS AND ANTENATAL CARE. This workshop for East and Southern African countries will take place in Kigali Rwanda in June 2017. In order to support the implementation of these guidelines, we would greatly appreciate the participation of your country in a pre-workshop survey.

The aim of the survey is to obtain a holistic picture of the situation of STI management and control in your country. Information from the survey will be used to advice the workshop and also further support addressing health system issues. We would like to recommend that the questionnaire be completed by a team of relevant responsible persons from the Ministry of Health (MOH), WHO, UNICEF and UNFPA in your country from each of the following areas;

1. Maternal and Newborn Health (MNH)
2. Prevention of Mother to Child Transmission of HIIV (PMTCT)
3. STI focal persons from (i) Sexual and Reproductive Health (SRH) and (ii) HIV programmes
4. Reproductive Health Commodity Procurement

We would appreciate if each question is responded to, indicating if information is not available where applicable and we welcome any additional comments that may help better reflect the situation.

We would be most grateful if you could complete the survey and submit your responses as soon as possible, but preferably no later than **11 April 2017** by email to Dr Morkor Newman on email [newmanm@who.int](mailto:newmanm@who.int) with copy to Dr Innocent Nuwagira on [nuwagirai@who.int](mailto:nuwagirai@who.int) ; Dr Tunde Adegboyega on [adegboyegaa@who.int](mailto:adegboyegaa@who.int) and Dr Nancy Kidula on [kidula@who.int](mailto:kidula@who.int)

Most sincere thanks for your kind support and cooperation

**COUNTRY………………………………………………………………………………………………………….**

**Kindly provide contact details of respondent team members below.**

| Name | Designation / Position | Institution / Organisation | Email | Phone |
| --- | --- | --- | --- | --- |
|  |  |  |  |  |
|  |  |  |  |  |
|  |  |  |  |  |
|  |  |  |  |  |
|  |  |  |  |  |
|  |  |  |  |  |
|  |  |  |  |  |
|  |  |  |  |  |
|  |  |  |  |  |
|  |  |  |  |  |

Part A: Sexually Transmitted Infections (STI) Landscape

**Management of STIs**

This section will assess the epidemiology of STIs and the response including planning, implementation, and monitoring of STI programmes.

**Burden of STIs:**

- Total number of cases in 2015 and 2016 (all types of STIs)
  - 2015………………………………………………
  - 2016………………………………………………
- Prevalence of STI in general population (from any population based survey)

| **Sexually transmitted disease** | **Prevalence** | **Survey/Source (DHS, AIS etc)** | **Year** |
| --- | --- | --- | --- |
| HIV |  |  |  |
| HSV2 |  |  |  |
| Syphilis |  |  |  |
| HPV |  |  |  |
| Other (specify) |  |  |  |

- Total cases by type /country classification of STIs in 2015/2016

(*Please provide data available from HMIS system depending on what is collected)*

| Disease classification | Total numbers | | Syndrome classification | Total numbers | |
| --- | --- | --- | --- | --- | --- |
|  | 2015 | 2016 |  | 2015 | 2016 |
| - Syphilis - Chlamydia - Gonorrhea - Herpes genitalis - Genital warts - Chancroid - Trichomoniasis - Other |  |  | - Genital ulcer disease - Urethral discharge (male) - Vaginal discharge - trichomoniasis - Pelvic Inflammatory disease (PID) |  |  |

**Management of Syphilis in Pregnancy:**

- Total No of pregnant women attending ANC1 in 2015……………………2016…………………………..
- Total No of pregnant women tested during ANC in 2015……………………2016………………………
- Total No of pregnant women testing positive for Syphilis during ANC in 2015…..……2016………….
- Total No of pregnant women testing positive who were treated for Syphilis during ANC in 2015……………………2016…………………………..

**Policy and governance:**

1. Existence of national strategy for prevention and control of STIs, possibly included in the Reproductive Health (RH) or HIV strategy or stand alone

( ) NO

( ) YES

Comment………………………………………………………………………………………………………………………………….

1a Which programme(s) provide overall coordination of the national STI prevention and control in your country


Comment………………………………………………………………………………………………………………………………….

1b what are the specific roles and responsibilities of each unit/department/programme in STI prevention and control in your country

| Department/unit/programme | Roles and responsibilities | Designation of focal point |
| --- | --- | --- |
| HIV - NACP |  |  |
| PMTCT |  |  |
| SRH |  |  |
| MNH |  |  |
| Oncology |  |  |
| NAC |  |  |
| Other (specify) |  |  |

1c In which department is the programme for **Elimination of Congenital Syphilis (ECS)** located/managed?

1. Existence of national guidelines for management of STIs?

( ) NO

( ) YES

If yes, indicate the year of the last update……………………………………Comments ………………………………….

1. Existence of national policy documents/ guidelines on prevention and management of congenital syphilis

( ) NO

( ) YES

If yes, indicate the document and the year of the last update…………………………………………….

Comment………………………………………………………………………………………………………………………………….

**Implementation strategy:**

1. Is there a functional monitoring and evaluation system in place for STIs?

( ) NO

( ) YES

Comment………………………………………………………………………………………………………………………………….

1. Are STI monitoring data integrated in the HMIS?

( ) NO

( ) YES

Comment………………………………………………………………………………………………………………………………….

5a which STI indicators are tracked in the HMIS system and how frequently?

| Indicator | Frequency of tracking |
| --- | --- |
|  |  |
|  |  |
|  |  |
|  |  |

5b list any other reporting systems or data sources for STI information

4. Is STI/Syphilis testing, prevention and care part of in the antenatal care package?

( ) NO

( ) YES

Comment………………………………………………………………………………………………………………………………….

6b. Is ANC syphilis testing using rapid diagnostic tests conducted by nurses and midwives in primary health care clinics?

( ) NO

( ) YES

Comment………………………………………………………………………………………………………………………………….

1. Is STI management **functionally integrated** into any of the following service delivery platforms?

| HIV comprehensive care | ( ) YES ( ) NO |
| --- | --- |
| Family planning services | ( ) YES ( ) NO |
| Adolescent friendly health services | ( ) YES ( ) NO |
| Postnatal care | ( ) YES ( ) NO |
| Others. Specify |  |

**Health workers capacity**

1. Is prevention and control of STIs addressed in the pre-service training curriculum for doctors, midwives and nurses?

( ) NO

( ) YES

Comment………………………………………………………………………………………………………………………………….

1. Does your country provide in-service training in the prevention and control of STIs for health workers in facilities?

( ) NO

( ) YES

Comment………………………………………………………………………………………………………………………………….

1. Existence of standardized national training materials for prevention and control of STIs?

( ) NO

( ) YES

If yes, indicate which training materials are available…………………………………………………………………..,

If yes indicate the dates of trainings and number of health workers trained in the matrix below

|  | 2015 | 2016 |
| --- | --- | --- |
| dates of trainings (months) |  |  |
| number of health workers trained |  |  |

10.b. Are midwives and nurses allowed to prescribe Benzathine penicillin?

( ) NO

( ) YES

Comment………………………………………………………………………………………………………………………………….

1. Is a supervision system in place for prevention and control of STIs?

( ) NO

( ) YES

Comment………………………………………………………………………………………………………………………………….

**Procurement and supply chain:**

1. Are essential drugs for management of STIs generally available?

( ) NO

( ) YES

Comment………………………………………………………………………………………………………………………………….

12.b. Is Benzathine penicillin included in the essential medicines list for PHC level?

( ) NO

( ) YES

Comment………………………………………………………………………………………………………………………………….

1. Are syphilis test kits generally available in primary health care clinics?

( ) NO

( ) YES

Comment………………………………………………………………………………………………………………………………….

1. Did the country use the dual test kits for HIV and syphilis for pregnant women in 2015 or 2016?

( ) NO

( ) YES

Comment………………………………………………………………………………………………………………………………….

Part B: Management of congenital syphilis and availability of Benzathine Penicillin

This part is designed to evaluate shortages of syphilis test kits and benzathine penicillin (BenPCN). This formulation of penicillin is used for the treatment of pregnant women with syphilis as it is the only known effective treatment to prevent congenital syphilis. Responses from this survey will be used to advocate for improvements in dual test kit and benzathine penicillin supply.

.

**Benzathine Penicillin stock out**

1. Was Benzathine penicillin available in your country in 2015 and 2016?

( ) NO (2015) ( ) NO (2016)

( ) YES (2015) ( ) YES (2016)

If yes, who provided resources for procurement and supply of Benzathine penicillin?

( ) Government

( ) Partners (please indicate which partners) ………………………………………………………………………………

2. was there a Benzathine penicillin stock out in 2015 or 2016 in your country (at the central level)?

( ) NO (2015) ( ) NO (2016)

( ) YES (2015) ( ) YES (2016)

If YES, when in 2015 and 2016

If NO, what were the total usable number of doses currently in storage at the central level at the end of 2015/ 2016 (1 dose = 2.4 million U?) 2015………………………………………2016…………………………………..

**Status of Benzathine Penicillin backlogs**

3. Did your country have any backlogged orders in 2015 or 2016?

( ) NO (2015) ( ) NO (2016)

( ) YES (2015) ( ) YES (2016)

If yes, what were the numbers of doses for all backlogged orders?

2015……………………………………………………………2016………………………………………………………………………………..

If yes, what were the expected delivery dates?

……………………………………………………………………………………………………………………………………………………………

**Procurement mechanisms for Benzathine Penicillin**

4. What mechanisms does your country regularly use to acquire Benzathine penicillin? [Mark all that apply, and use the box below for more detailed answers]

( ) There is no centralized acquisition of Benzathine Penicillin in my country

( ) National bid

( ) International bid

( ) Procurement agreements with a UN or any other Agency

Please specify which agency is used for procurement, or share any other relevant comments on this issue in the space below:

|  |
| --- |

**Alternative procurement mechanisms used to manage shortage**

5. If there was a shortage in your country, did your country use alternative mechanisms to acquire Benzathine penicillin? [Mark all that apply, and use the box below for more detailed answers. If there was no shortage of Benzathine Penicillin in country, proceed to Q 6

( ) NO, my country has NOT used alternative mechanisms to acquire Benzathine Penicillin

( ) YES, my country HAS used alternative mechanisms to acquire Benzathine Penicillin

If YES, please indicate which mechanisms below

|  |
| --- |

**Average monthly consumption**

6. What has been the average monthly consumption of Benzathine Penicillin doses in 2015 and 2016 in your country? 2015…………………………………………………2016………………………………………………………………….

7. Based on your country's projected need for Benzathine Penicillin and current supply, what was the estimated Benzathine Penicillin shortfall in total number of doses for and 2016?

**2015** **……………………………………………………………2016……………………………………………………………………..**

**Estimated need to treat pregnant women**

8. If available, please provide the estimated number of doses needed to treat pregnant women with syphilis in 2015 and 2016? ………………………………………………………………………………………………………………………

**Insight for the shortage of Benzathine Penicillin**

9. Please provide insight as to whether any of the following reasons for a shortage of Benzathine Penicillin applies in your country. [Mark all that apply, and use the box below for more detailed answers] If there is no shortage of Benzathine penicillin in country, please proceed to question 10.

( ) There is a backlog of orders

( ) Exceptional increased demand for Benzathine Penicillin

( ) Funding not available in country for purchase of Benzathine Penicillin

( ) No manufacturer available for purchase

( ) No distributers available for purchase

Are there any reasons not mentioned above for the Benzathine Penicillin shortage in your country? (Please specify):

|  |
| --- |

Is Benzathine penicillin provided free of charge for syphilis positive pregnant women in your country?

( ) NO

( ) YES

If no, approximately how much does one dose cost in the public sector?.............................................

**Other relevant information on Benzathine penicillin shortages**

10. If your country experienced or is experiencing Benzathine penicillin shortage, please provide insights to the following questions below to help WHO/AFRO better understand the problem:

10a. Please list the issues your country is facing due to the shortage (i.e. purchased Benzathine penicillin at higher cost; in different presentation; others):

|  |
| --- |

10b. Please list the perceived causes for Benzathine penicillin shortage:

|  |
| --- |

10c. Please list actions taken by the country to minimize problems due to the Benzathine penicillin shortage:

|  |
| --- |

10d. Please list desired solutions for Benzathine penicillin shortage:

|  |
| --- |

**Any other comments or suggestions**

**11. Please provide any additional comments to improve the STIs management.**

For example, we will be interested to hear about the acceptability and utilization of Benzathine Penicillin.

|  |
| --- |
