# Supplementary material for: Shortages of benzathine penicillin for prevention of mother-to-child transmission of syphilis: An evaluation from multi-country surveys and stakeholder interviews
Source: PLoS Med. 2017 Dec 27;14(12):e1002473. doi: 10.1371/journal.pmed.1002473 (PMC5744908; doi:10.1371/journal.pmed.1002473)
Supplement: S5 Appendix — (DOCX) [file pmed.1002473.s005.docx]

**Enquête sur la gestion des Infections Sexuellement Transmissibles (ISTs), y compris la syphilis congénitale et la disponibilité de la Benzathine Penicilline dans les pays de la Région Africaine de l’OMS.**

**Background:**

Les points focaux de la santé maternelle et ceux en charge de la gestion des ISTs au niveau des ministères de la santé et des bureaux pays de l’OMS sont invités à participer à un atelier régional de dissémination des nouvelles directives de l’OMS (2016) pour le traitement des infections sexuellement transmises et les soins prénatals qui aura lieu à Ouagadougou, au Burkina Faso, du 15 au 17 novembre 2016. Afin de soutenir la mise en œuvre de ces nouvelles directives dans les pays, nous vous saurons gré de bien vouloir participer à cette enquête pré-atelier en répondant aux questions ci-dessous. Pour chaque question, n’hésitez pas à ajouter des commentaires si nécessaire.

Nous vous prions de répondre aux questions ci-dessous et faire parvenir les réponses de votre pays le plus tôt possible et au plus tard le 11 novembre 2016 aux personnes suivantes :

Dr Ouedraogo Leopold: [ouedraogol@who.int](mailto:ouedraogol@who.int); Dr Bigirimana Françoise: [bigirimanaf@who.int](mailto:bigirimanaf@who.int); et Dr Sanni Saliyou: [sannis@who.int](mailto:sannis@who.int)

**Section A: Gestion des ISTs**

Cette section est conçue pour évaluer le processus de planification, de mise en œuvre et de suivi des programmes de lutte contre les ISTs en général.

**Ampleur des ISTs:**

- Nombre de cas cumulés en 2015 (tous types d’ISTs)
- Nombre de cas par type d’IST en 2015
- Nombre total de femmes enceintes dépistées positives pour la Syphilis en CPN en 2015

**Politique:**

1. Existence de stratégie nationale de prévention et de contrôle des ISTs, probablement intégrée dans la stratégie/programme SR ou VIH

( ) NON

( ) OUI

1. Existence de directives nationales pour la lutte contre les ISTs?

( ) NON

( ) OUI

Si oui, quelle est la date de la dernière mise à jour?

1. Existence de documents nationaux /directives sur la prévention et la gestion de la syphilis congénitale

( ) NON

( ) OUI

Si oui, indiquer le document et l’année de la dernière mise à jour?

**Mise en œuvre de la stratégie:**

1. Est-ce qu’il existe un système fonctionnel de notification et de suivi-évaluation des ISTs?

( ) NON

( ) OUI

1. Est-ce que les données de notification et de suivi sont intégrées dans le SNIS (Système National d’Information Sanitaire)?

( ) NON

( ) OUI

1. Est-ce que la Syphilis est prise en compte dans le paquet d’interventions de la Consultation Prénatale Recentrée (CPNR)?

( ) NON

( ) OUI

**Capacité des agents de santé**

1. Est-ce que la formation de base et en cours d’emploi pour la prévention et le contrôle des ISTs est effective dans votre pays? Veuillez préciser s’il vous plaît.

( ) NON

( ) OUI

1. Existence de matériels de formation pour la prévention et le contrôle des IST?

( ) NON

( ) OUI

Si oui, indiquer les matériels disponibles, les dates des formations réalisées et le nombre d’agents de santé formés durant la période 2015-2016

1. Est-ce qu’un système de supervision de la prévention et du contrôle des IST est en place ?

( ) NON

( ) OUI

**Chaine d’approvisionnement:**

1. Disponibilité des médicaments essentiels pour la gestion des ISTs?

( ) NON

( ) OUI

1. Disponibilité des kits de dépistage de la syphilis?

( ) NON

( ) OUI

1. Est-ce que le pays utilise les tests de dépistage combiné du VIH et de la syphilis chez les femmes enceintes?

( ) NON

( ) OUI

**Section B: Gestion de la syphilis congénitale et disponibilité de la Benzathine Penicilline**

Cette section est conçue pour évaluer les ruptures de stock des kits de dépistage de la syphilis ainsi que de la benzathine penicilline. Cette forme de pénicilline retard est utilisée pour le traitement de la syphilis chez la femme enceinte, car c’est le seul traitement efficace connu pour prévenir la syphilis congénitale. Les résultats de cette enquête seront utilisés pour faire le plaidoyer pour l’amélioration de l’approvisionnement en kits de dépistage combiné du VIH et de la syphilis ainsi que de la benzathine penicilline.

**Ruptures de stock de Benzathine Penicilline (**BenPCN)

1. Est-ce que la BenPCN est disponible dans le pays?

( ) NON

( ) OUI

Si oui, d’où proviennent les ressources pour l’offre de la BenPEC ?

( ) Gouvernement

( ) Partenaires (prière préciser quels partenaires)

2. Est-ce qu’actuellement il y a une rupture de stock de BenPCN dans le pays (au niveau central)?

( ) NON

( ) OUI

Si OUI, depuis quand?

Si NON, quel est le nombre total de doses actuellement en stock au niveau central (1 dose = 2.4 million IU?)

**Etat des commandes non exécutées de Benzathine Penicilline**

3. Est-ce que votre pays a des commandes non encore livrées (en attente)?

( ) NON

( ) OUI

Si oui, à quelle quantité de doses correspondent toutes les commandes non encore livrées?

Si oui, quelles sont les dates attendues de livraison?

**Mécanismes d’approvisionnement en Benzathine Penicilline (**BenPCN)

4. A travers quels mécanismes/circuits le pays s’approvisionne-t-il régulièrement en BenPCN? [Cocher tout ce qui s’applique, et utiliser l’espace ci-dessous pour donner des réponses plus détaillées]

( ) Il n’y a pas de mécanisme centralisé d’approvisionnement en BenPCN dans mon pays

( ) Appel d’offre National

( ) Appel d’offre International

( ) Accord d’approvisionnement avec une agence du SNU

Prière préciser l’agence du SNU concerné, ou faire des commentaires appropriés dans l’espace ci-dessous:

**Mécanismes alternatifs d’approvisionnement utilisés pour pallier aux ruptures de stocks**

5. Si votre pays connaît (ou a connu) une rupture de stock, est-ce que des mécanismes alternatifs sont (ou ont été) utilisés pour s’approvisionner en BenPCN? [Cocher tout ce qui s’applique, et utiliser l’espace ci-dessous pour donner des réponses plus détaillées]

( ) Il n’y a pas de rupture de stock de BenPCN dans le pays

( ) NON, mon pays n’a pas utilisé de mécanismes alternatifs

( ) OUI, mon pays a utilisé des mécanismes alternatifs

Si OUI, préciser les mécanismes

**Consommation Moyenne Mensuelle**

6. Quelle a été la moyenne de consommation mensuelle de doses de BenPCN dans votre pays EN 2015?

7. Sur la base des estimations de besoins du pays et du niveau actuel de l’offre, quelle est la quantité estimée (en doses) des besoins non satisfaits en BenPCN pour 2016?

**Estimations des besoins pour traiter les femmes enceintes**

8. Si disponible, quel est l’estimation du nombre de doses nécessaires pour traiter la syphilis chez les femmes enceintes en 2016?

**Informations sur les besoins non satisfaits de BenPCN**

9. Parmi les raisons suivantes pouvant justifier les besoins non-satisfaits de BenPCN, prière indiquer celles qui s’appliquent à votre pays. [Cocher tout ce qui s’applique, et utiliser l’espace ci-dessous pour donner des réponses plus détaillées]:

( ) Il n’y a pas de besoins non satisfaits de BenPCN dans le pays

( ) Il y a des commandes non encore livrées

( ) Accroissement exceptionnel de la demande de BenPCN

( ) Non disponibilité de fonds dans le pays pour l’achat de la BenPCN

( ) Il n’y a aucun fabriquant auprès duquel on peut acheter la BenPCN

( ) Il n’y a aucun distributeur auprès duquel on peut acheter la BenPCN

Est-ce qu’il existe d’autres raisons non mentionnées ci-dessus qui pourraient aussi justifier les besoins non-satisfaits de BenPCN dans votre pays ? (Prière préciser):

**Autres informations pertinentes sur les besoins non-satisfaits de BenPCN**

10. Si votre pays fait face à des besoins non satisfaits de BenPCN, prière répondre aux questions ci-dessous pour aider l’OMS/AFRO à mieux comprendre le problème:

10a. Prière citer les défis auxquels votre pays est confronté du fait des besoins non satisfaits de BenPCN (i.e. achat de BenPCN à un prix élevé; sous différentes formes pharmaceutiques; autres):

10b. Prière citer les causes perçues des besoins non satisfaits de BenPCN:

10c. Prière citer les actions prises jusqu’à présent par le pays pour minimiser les problèmes liés aux besoins non satisfaits de BenPCN:

10d. Prière citer les solutions proposées aux problèmes liés aux besoins non satisfaits de BenPCN:

**Any other comments or suggestions**

11. **Veuillez s’il vous plaît donner des commentaires additionnels pour revitaliser la lutte contre les ISTs.**

A titre d’exemple, nous serions intéressés d’avoir de plus amples informations sur l’acceptabilité et l’utilisation de la BenPCN.

**Adresse complète des Répondants**

12. Prière fournir l’adresse de la personne responsable de la compilation des réponses aux questions de cette enquête:

Nom:

Fonction:

Institution:

Pays:

e-mail:

Téléphone:
