# Supplementary material for: Shortages of benzathine penicillin for prevention of mother-to-child transmission of syphilis: An evaluation from multi-country surveys and stakeholder interviews
Source: PLoS Med. 2017 Dec 27;14(12):e1002473. doi: 10.1371/journal.pmed.1002473 (PMC5744908; doi:10.1371/journal.pmed.1002473)
Supplement: S6 Appendix — (DOCX) [file pmed.1002473.s006.docx]

***WHO’s*** *email survey used for CDC country directors*

Dear PEPFAR country director,

I am a CDC Med-Epi at WHO in the Department of Reproductive Health.

We have received multiple anecdotal reports from several countries (some PEPFAR) of shortages/stockouts of benzathine penicillin, the first line treatment option for pregnant women with syphilis and infants born with congenital syphilis.

We are asking country directors to share any information on current or recent benzathine penicillin shortages.

If you are experiencing a shortage of benzathine penicillin in Ethiopia, this quick survey will help us understand the context.  Thank you in advance for completing or forwarding to your appropriate staff.

<https://www.surveymonkey.com/r/62XKXYR>

If you are not or have not experienced a shortage of benzathine penicillin, kindly let us know this as well.  Your help is greatly appreciated as we try to uncover the sites and sources of the shortages such that we can respond appropriately.

Thank you for your reply.

Kind regards,

Melanie

Melanie Taylor MD, MPH

CDC Medical Officer

WHO Department of Reproductive Health and Research

20 Avenue Appia

Geneva, Switzerland

[mtaylor@who.int](mailto:mtaylor@who.int)  or [MDT7@cdc.gov](mailto:MDT7@cdc.gov)

Ph: +41-227-912-172

**To the reviewer: Only 1 PEPFAR country representative completed the survey monkey survey. The remaining PEPFAR representatives simply replied to the email with a response of having or not having a benzathine penicillin shortage.**
