# Supplementary material for: Shortages of benzathine penicillin for prevention of mother-to-child transmission of syphilis: An evaluation from multi-country surveys and stakeholder interviews
Source: PLoS Med. 2017 Dec 27;14(12):e1002473. doi: 10.1371/journal.pmed.1002473 (PMC5744908; doi:10.1371/journal.pmed.1002473)
Supplement: S8 Appendix — (DOCX) [file pmed.1002473.s008.docx]

**Benzathine Penicillin G (BPG) – Proposed discussion guide**

**[DISTRIBUTORS]**

Clinton Health Access Initiative –July 2016

**Quality Questions**

- Is [DISTRIBUTOR] auditing API and/ or final formulation for BP?

- What is the [DISTRIBUTOR]’s quality standard that [DISTRIBUTOR] is working toward (API or final formulation) for BP?
- Which API manufacturers and/or Final Formulators (FF) have [DISTRIBUTOR] been audited by [DISTRIBUTOR]? When did these audits take place and what is typical frequency for auditing?
- What were relative strengths and weakness of the API manufacturer or FF? Have there been any quality concerns arising from the audits?
- Have buyers reported any product quality concerns back to [DISTRIBUTOR] for BP? Is there a process for this?
- Have there been any supply constraints? If yes, how has [DISTRIBUTOR] tried to address any quality issues that have led to supply constraints?

**Ordering Questions**

- What is minimum order quantity (MOQ) required for each of the FF? Does this differ by strength (e.g., 0.6M vs 1.2M vs 2.4M)?
- What is their average indicative delivery lead-time? What degree of flexibility is there in delivery dates?
- Is it possible to negotiate shorter lead-times? If yes, under what conditions?
- Is it possible to re-schedule orders after being placed? If yes, at what stage and how often?
- Is it possible to cancel orders? If yes, under what conditions?
- What is the average level of delivery compliance with quantities ordered and scheduled delivery dates?
