# Supplementary material for: Shortages of benzathine penicillin for prevention of mother-to-child transmission of syphilis: An evaluation from multi-country surveys and stakeholder interviews
Source: PLoS Med. 2017 Dec 27;14(12):e1002473. doi: 10.1371/journal.pmed.1002473 (PMC5744908; doi:10.1371/journal.pmed.1002473)
Supplement: S9 Appendix — (DOCX) [file pmed.1002473.s009.docx]

**Benzathine Penicillin G (BPG) – Proposed discussion guide**

**[PURCHASERS/MoH STAFF/NATIONAL LEADS]**

Clinton Health Access Initiative –July 2016

**Questions**

- Introductions
- Purpose of the call
- Understand current hypothesis on BP stock outs
  - Therefore, can you provide an overview of the work that your group has done on benzathine penicillin?
- What drove you all to undertake [specific work that partner has conducted]?
- What emerged as the key drivers of benzathine penicillin shortages and stock outs [rom the specific work that your group has done]?
- Who are the API manufactures:
  - Who is GMP approved? Are there potential API manufacturers in [region]? What would it take to get them GMP or SRA approved?
  - Are there [region] API manufacturers that have capacity to increase production?
  - What have been the impacts of GMP non-compliance? We know that 1 API manufacturer has been non-compliant and heard possibly that another has? Can you comment on this?
  - Have you seen any other emerging API suppliers?
- What is your visibility of the current supply chain?
- Do countries have a perception that there will be continued BP shortfall?
- What is preventing accurate forecast? How can countries improve their forecasts? What can be done to improve on the volatility of demand?
- What do you see as the most important and urgent next steps? If you were to follow up on learnings from the work that you have done, what would you like to further understand from the supplier perspective? From the demand perspective?
- What role do you see [your organization] playing in these next steps?
- Where should CHAI focus its analysis?
