# Supplementary material for: Shortages of benzathine penicillin for prevention of mother-to-child transmission of syphilis: An evaluation from multi-country surveys and stakeholder interviews
Source: PLoS Med. 2017 Dec 27;14(12):e1002473. doi: 10.1371/journal.pmed.1002473 (PMC5744908; doi:10.1371/journal.pmed.1002473)
Supplement: S10 Appendix — (DOCX) [file pmed.1002473.s010.docx]

**Benzathine Penicillin G (BPG) – Proposed discussion guide [MANUFACTURER]**

Clinton Health Access Initiative –July 2016

**High level questions**

- Has [Manufacturer] experienced disruptions to its supply of either BPG API and/or Finished Products? If no, is [Manufacturer] aware of other market disruptions and what is known?
- How relatively attractive is the BPG API and/or Finished Product market for [Manufacturer]? How does production capacity and commercial opportunity for BPG fit within priorities? What changes would make this market more commercially attractive for [Manufacturer]?
- What are the market dynamics for BPG? How fragmented or concentrated is demand – both in terms of numbers of major buyers and levels of commercial intermediaries?
- What are [Manufacturer]’s plans for the BPG market? What changes, if any, does this reflect from previous strategy? What factors are influencing [Manufacturer]’s BPG strategy?

**Detailed guiding questions**

1. **Technical: capacity and assets**

- What type(s) of BPG product(s) (both API and/or final formulations) does [Manufacturer] currently manufacturing? How are these manufactured and which components are made in-house?
- For which markets is/are this (these) product(s) being manufactured?
  - Human vs. veterinary markets?
  - Domestic vs. international markets? If international, which regions predominantly?
  - Percentage sold as API vs. final formulation

1.1. BPG API manufacturing (if applicable)

- How is production capacity allocated? Is there dedicated BPG production?
- What is the estimated utilization of BPG production capacity? How does this compare to the demand for your product?
- What is your average indicative delivery lead-time?
- Do orders typically come at the same time or spread out over the year?
- Are there minimum order quantities?
- What is the product specifications?
- What is the average indicative price/margin?

1.2. Fill/Finishing (if applicable)

- How is production capacity allocated? Is there dedicated BPG production?
- What percentage of demand for F/F capacity does BPG represent?
- What is your average indicative delivery lead-time for finished product?
- Do orders typically come at the same time or spread out over the year?
- Are there minimum order quantities?

1. **Perspective on the market**

- In the past 3 to 5 years, 1) how has demand evolved and 2) has the market changed?
- What trends do you see related to pricing and buyer purchasing behavior for API and/or F/F product?
- Who are your key customers? (countries, global buyers, formulators, …)
- Is there significant variation between customers in terms of quality demands, volumes purchased?
- What makes BPG commercially attractive or unattractive?
- What are your future market perspectives? What risks and opportunities do you see?

1. **Perspective on market shortages**

- Has [Manufacturer], at any point in the past, had disruptions to its BPG supply chain?
  - Which products and countries were affected? What was the root cause?  How was this managed and/or communicated?
- Is there a risk that you will not be able to supply in the future?
- Is there a future risk mitigation plan?
- Would you have any suggestions on how the risk for supply constraints could be reduced?
