# Supplementary material for: Shortages of benzathine penicillin for prevention of mother-to-child transmission of syphilis: An evaluation from multi-country surveys and stakeholder interviews
Source: PLoS Med. 2017 Dec 27;14(12):e1002473. doi: 10.1371/journal.pmed.1002473 (PMC5744908; doi:10.1371/journal.pmed.1002473)
Supplement: S12 Appendix — (DOCX) [file pmed.1002473.s012.docx]

**Benzathine Penicillin G (BPG) Market Assessment**

**Current state of finished dosage formulations (FDF) suppliers for BPG**

Clinton Health Access Initiative –August 2016

The following represents CHAI’s assessment of FDFs currently active in the market. This information was collected from readily available company product lists available online as of August 2016 and may not be comprehensive of the full market.

**Branded BPG Product Available, Brand Name (Manufacturer)**

| - Bencelin (Antibioticos de Mexico) |
| --- |
| - Benzetacil (Eurofarma Laboratorios) |
| - Bepeben (Teuto-Brasileiro Laboratorio) |
| - Bicillin LA (Pfizer) |
| - Cepacilina (Reig Jofre) |
| - Lentocilin (Atral Laboratorios) |
| - Longacillin (Hindustan Antibiotics) |
| - Pen di Ben (Bago Laboratorios) |
| - Penadure, Penidural (Wyeth/ Pfizer) |
| - Pencom (Alembic) |
| - Pendepon (Biotika Bohemia) |
| - Tardocillin (Infectopharm) |
| - Unicil L-A (Unipharm de Mexico) |

**Generic BPG Product Available, Name (Manufacturer)**

| - BPG (CSPC) |
| --- |
| - BPG (Ethiopian Manufacturing) |
| - BPG (Jiangxi Dongfeng) |
| - BPG (Karnataka) |
| - BPG (NCPC) - BPG (PanPharma) |
| - BPG (Phapros) |
| - BPG (Reyoung) |
| - BPG (Sinochem) |

**BPG Products Exited market or Unclear if marketed, Name (Manufacturer)**

| - Ampiretard (CIBRAN Companhia Bras.) |
| --- |
| - Benacilina (IVAX) |
| - Bencilpenicilina Benzatina (Volta Labo) |
| - Benzafur (IVAX) |
| - Benzanil Compuesto (Roche) |
| - Benzanil Simple (Roche) |
| - Benzapen (Teuto-Brasileiro Labo) |
| - Benzatron (Ariston Industrias) |
| - Benzycillin-3, 5 (Kraspharma) |
| - Bicillin -1, 3, 5 (Sintez) |
| - Depotpen (GlaxoSmithKline) |
| - Diaminocillina (Fournier) |
| - Durabiotic (Sandoz; Teva) |
| - Extencilline (Sanofi-Aventis) |
| - Karbasalin (Chile Laboratorios) |
| - Kitapen (Dansk-Flama Instituto) |
| - Lentopenil (Grossman Laboratorios) |
| - Liademycin Retard (Abbott Labs) |
| - Longacilin (Biolab Sanus Farma) |
| - Neo Benzil (Neo Quimica Comercio) |
| - Neocepacilina (CEPA Schwartz ) |
| - Normabenzil (Farmoquimica) |
| - Pencil B (Laboratorio Farmaceutico) |
| - Pendysin (Jenapharm) |
| - Penextilline (Sanofi-Aventis) |
| - Penicilina Benzatina (Chile Labo) |
| - Penilente LA (Novo Nordisk) |
| - Penilevel Retard (ERN Laboratorios) |
| - Peniroger Retard (UCB Pharma) |
| - Pipercilina (Urca, Spain) |
| - Provipen (Generfarma) |
| - Retacillin Compositum (Jenapharm) |
| - Retarpen (Novartis; Biochemie) |
| - Silcopen (Valmor Laboratorios) |
| - Tri-Wycillina (Pfizer) |
| - Ultracillin (Caps Pharmaceuticals) |
