# Supplementary material for: Shortages of benzathine penicillin for prevention of mother-to-child transmission of syphilis: An evaluation from multi-country surveys and stakeholder interviews
Source: PLoS Med. 2017 Dec 27;14(12):e1002473. doi: 10.1371/journal.pmed.1002473 (PMC5744908; doi:10.1371/journal.pmed.1002473)
Supplement: S7 Appendix — (DOCX) [file pmed.1002473.s007.docx]

***Response to reviewers: Please find below the list of countries surveyed by region. Responding countries are noted by a “1” in column 3 “Response”. Countries experiencing shortages are designated by a “1” in column 4 “Shortage”***

Table #: Countries surveyed regarding benzathine penicillin supply

| **Country** | **WHO Region** | **Response 1 = Yes** | **Shortage 1 = Yes** |
| --- | --- | --- | --- |
| Angola | AFRO | 1 | 0 |
| Anguilla | PAHO | 0 | 0 |
| Antigua and Barbuda | PAHO | 1 | 0 |
| Argentina | PAHO | 0 | 0 |
| Bahamas | PAHO | 1 | 0 |
| Bangladesh | SEARO | 0 | 0 |
| Barbados | PAHO | 1 | 0 |
| Belize | PAHO | 0 | 0 |
| Bermuda | PAHO | 1 | 0 |
| Bolivia | PAHO | 0 | 0 |
| Botswana | AFRO | 1 | 0 |
| Burkina Faso | AFRO | 0 | 0 |
| Burma-Myanmar | SEARO | 1 | 0 |
| Burundi | AFRO | 0 | 0 |
| Cayman Islands | PAHO | 1 | 0 |
| Central African Rep | AFRO | 0 | 0 |
| Chad | AFRO | 1 | 0 |
| Chile | PAHO | 0 | 0 |
| Columbia | PAHO | 0 | 0 |
| Costa Rica | PAHO | 0 | 0 |
| Cote D'Ivoire | AFRO | 0 | 0 |
| Cuba | PAHO | 0 | 0 |
| Dominica | PAHO | 1 | 0 |
| Dominican Republic | PAHO | 0 | 0 |
| DRC | AFRO | 0 | 0 |
| Ecuador | PAHO | 1 | 0 |
| El Salvador | PAHO | 0 | 0 |
| France | EURO | 0 | 0 |
| French Guiana | PAHO | 1 | 0 |
| Gabon | AFRO | 1 | 0 |
| Gambia | AFRO | 0 | 0 |
| Grenada | PAHO | 0 | 0 |
| Guadelupe | PAHO | 1 | 0 |
| Guatemala | PAHO | 0 | 0 |
| Guyana | PAHO | 0 | 0 |
| Haiti | PAHO | 0 | 0 |
| Honduras | PAHO | 0 | 0 |
| Japan | WPRO | 0 | 0 |
| Kenya | AFRO | 0 | 0 |
| Korea | WPRO | 0 | 0 |
| Lesotho | AFRO | 0 | 0 |
| Mali | AFRO | 1 | 0 |
| Martinique | PAHO | 1 | 0 |
| Mauritania | AFRO | 0 | 0 |
| Mexico | PAHO | 0 | 0 |
| Mongolia | WPRO | 0 | 0 |
| Montserrat | PAHO | 0 | 0 |
| Morrocco | AMRO | 0 | 0 |
| Mozambique | AFRO | 1 | 0 |
| Nicaragua | PAHO | 0 | 0 |
| Niger | AFRO | 0 | 0 |
| Papua New Guinea | WPRO | 0 | 0 |
| Paraguay | PAHO | 0 | 0 |
| Peru | PAHO | 0 | 0 |
| Portugal | EURO | 0 | 0 |
| Rwanda | AFRO | 0 | 0 |
| Senegal | AFRO | 0 | 0 |
| South Sudan | AFRO | 0 | 0 |
| Spain | EURO | 0 | 0 |
| St. Kitts and Nevis | PAHO | 1 | 0 |
| St. Lucia | PAHO | 0 | 0 |
| St. Vincent and the Grenadines | PAHO | 0 | 0 |
| Swaziland | AFRO | 1 | 0 |
| Tanzania | AFRO | 0 | 0 |
| Thailand | SEARO | 0 | 0 |
| Togo | AFRO | 0 | 0 |
| Turks and Caicos Islands | PAHO | 1 | 0 |
| Ukraine | EURO | 1 | 0 |
| United Kingdom | EURO | 0 | 0 |
| Uruguay | PAHO | 0 | 0 |
| Venezuela | PAHO | 0 | 0 |
| Australia | WPRO | 0 | 1 |
| Benin | AFRO | 0 | 1 |
| Brazil | PAHO | 0 | 1 |
| Cambodia | SEARO | 0 | 1 |
| Cameroon | AFRO | 0 | 1 |
| Canada | PAHO | 0 | 1 |
| Congo | AFRO | 0 | 1 |
| Ethiopia | AFRO | 0 | 1 |
| Germany | EURO | 0 | 1 |
| Ghana | AFRO | 0 | 1 |
| Guinea | AFRO | 0 | 1 |
| India | SEARO | 0 | 1 |
| Indonesia | SEARO | 0 | 1 |
| Jamaica | PAHO | 0 | 1 |
| Liberia | AFRO | 0 | 1 |
| Madagascar | AFRO | 0 | 1 |
| Malawi | AFRO | 0 | 1 |
| Namibia | AFRO | 0 | 1 |
| Netherlands | EURO | 0 | 1 |
| New Zealand | WPRO | 0 | 1 |
| Nigeria | AFRO | 0 | 1 |
| Panama | PAHO | 0 | 1 |
| Philippines | WPRO | 0 | 1 |
| Sierra Leone | AFRO | 0 | 1 |
| South Africa | AFRO | 0 | 1 |
| Suriname | PAHO | 0 | 1 |
| Trinidad and Tobago | PAHO | 0 | 1 |
| Uganda | AFRO | 0 | 1 |
| United States | PAHO | 0 | 1 |
| Zambia | AFRO | 0 | 1 |
| Zimbabwe | AFRO | 0 | 1 |
| Greece | EURO | 0 | 1 |
| Bulgaria | EURO | 0 | 0 |
| Cyprus | EURO | 0 | 0 |
| Poland | EURO | 0 | 0 |
| Croatia | EURO | 0 | 1 |
| Romania | EURO | 0 | 0 |
| Switzerland | EURO | 0 | 1 |
